# Mineralogy and uranium leaching of ores from Triassic Peribaltic sandstones

**DOI:** 10.1007/s10967-014-3362-0

**Published:** 2014-08-01

**Authors:** Dorota Gajda, Katarzyna Kiegiel, Grazyna Zakrzewska-Koltuniewicz, Ewelina Chajduk, Iwona Bartosiewicz, Stanislaw Wolkowicz

**Affiliations:** 1 Institute of Nuclear Chemistry and Technology, Dorodna 16, 03-195 Warsaw, Poland; 2 Polish Geological Institute National Research Institute, Rakowiecka 4, 00-975 Warsaw, Poland

**Keywords:** Uranium, Sandstones, Acid leaching, Alkaline leaching

## Abstract

The recovery of uranium and other valuable metals from Polish Peribaltic sandstones were examined. The solid–liquid extraction is the first stage of the technology of uranium production and it is crucial for the next stages of processing. In the laboratory experiments uranium was leached with efficiencies 71–100 % by acidic lixiviants. Satisfactory results were obtained for the alkaline leaching process. Almost 100 % of uranium was leached with alkaline carbonate solution. In post leaching solutions only uranium and small amounts of vanadium were present.

## Introduction

Uranium plays an important role in generation of nuclear power. As a key substance for production of the fuel for nuclear reactors uranium, more common element in the earth’s crust occurring in rocks, soil, rivers and ocean waters, has to be extracted from the raw material in complex hydrometallurgical process involving many separation steps. Uranium in the ore often is accompanied by other rare metals that can be recovered in technological process to improve the profitability of the whole venture.

The characteristics of the material originating from uranium ores vary significantly from deposit to deposit. The effect of ore mineralogy and mineral liberation on the leaching behaviour of uranium is not well defined. The procedure of uranium extraction must be designed to fit specific characteristics of the ore; however the general scheme of the process is similar for most of the ore materials. The basic steps of processing of uranium ores are crushing and grinding, leaching, solid–liquid separation, ion exchange or solvent extraction, and finally precipitation of the final product, yellow cake—U_3_O_8_ [1, 2]. Tetravalent uranium has low solubility in both the acid and in carbonate solutions. For this reason, the first step in uranium leaching process is oxidation of uranium to uranium(VI) form. The use of oxidants e.g. manganese oxide, sodium chlorate or hydrogen peroxide, increases the leaching ability of uranium in water.

Leaching with sulphuric acid is the predominant process for recovery of uranium from the rocks [3–5]. Typically, leaching recovery with H_2_SO_4_ ranges from 85 to 95 %. However, this method is not economical for the carbonate materials due to their high acid consumption. This kind of ores requires alkaline processing technology for the recovery of uranium [6, 7]. In comparison with acid processing, alkaline leaching has the advantage of being selective for uranium. Uranium was selectively leached by the mixture of sodium carbonate, sodium hydroxide and hydrogen peroxide from hydrous oxide Egyptian monazite [8]. This method led to obtaining uranium of purity not <99 %. Similarly, the leaching of Polish sandstone ores by using the oxidative process for SIMFUEL [9] was successful. The almost complete extraction of uranium was observed in that case [10].

The arsenic-uranium ore was leached by 3 M ferric chloride [11]. This process gave approximately 92 % of uranium and arsenic precipitated as ferric arsenate. Enhancement in the leaching rate of uranium compared to conventional mechanical agitation was observed in sono-chemical leaching [12]. Bioleaching, lower energy consuming and environmental-friendly method, was used with success for leaching low-grade uranium ores [13].

In the present work the leaching behaviour of uranium from sandstones has been studied. Both procedures, acidic and alkaline, were tested to obtain high efficiency of leaching and good uranium separation. ICP-MS analysis was applied to determine the total uranium content in post-leaching solutions. This analytical technique is favourable since it enables to measure directly the mass concentration of total uranium without any chemical separation. It allows also analysing the content of big variety of other metals that accompany uranium in the ore.

## Materials and methods

### Geological-mineralogical characterization

The studied uranium mineralization is hosted by the Lower Triassic Upper Bunter sandstones occurring in area east of Gdańsk, central part of the Peribaltic Syneclise. The highest concentrations of uranium appear related to gray and gray-greenish poorly diagnosed, fine-grained sandstones. These rocks were deposited in environment of braided and meandering rivers and a shallow brackish reservoir. Source areas of clastic material of these rocks were situated in the north, in areas of the present day Baltic and southern Sweden. The recorded mineralization is assigned to sandstone deposits of the tabular and blanket types in the classification scheme of Dahlkamp [14, 15]. Deposits of these types are characterized by high vertical and horizontal variability in content of uranium, which implies problems in assessing their geological resources. Two ore bodies, named Krynica Morska and Ptaszkowo, were identified in the studied area. However, these bodies are situated at depths of at least 750 m so they have to be treated as prognostic or perspective [16].

Uranium deposits of the sandstone type are usually characterized by increased concentrations of V, Se, Mo, Pb, As, Ag and other metals. These metals occur in the form of aureoles around the uranium mineralization, which sometimes overlap geometrically one another. Thorium, often associated with accessory heavy minerals, is not present here due to epigenetic nature of this uranium mineralization.

**Fig. 1:**
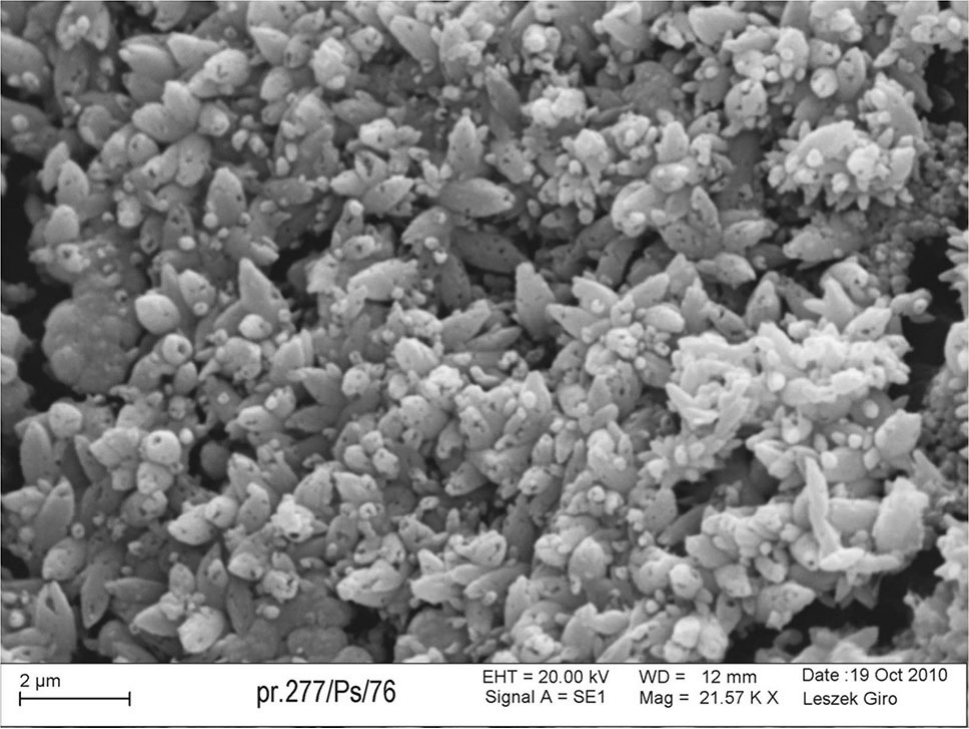
Uranium mineralization in the form of rhomboedric crystals (probably coffinite) in the highly porous sandstones with carbonate cement

**Fig. 2:**
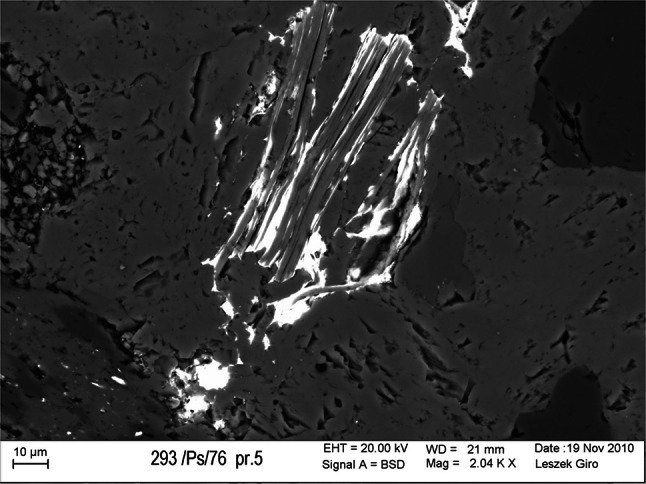
Uranium mineralization fills spaces between micas crystals

Scanning electron microscopy (SEM) combined with energy dispersive X-ray analysis (EDX) showed that uranium mineralization occurs here in amorphous or very finely crystalline (nasturane) and finely crystalline (coffinite) forms (Fig. 1). It occurs in the pore space between grains of clastic rocks or healing of micro-fractures. Uranium is also found to be absorded by illite and other clay minerals (Fig. 2). Concentrations of uranium also infill fractures and voids in quartz, pyrite, carbonates and other minerals which form cement, which indicates a few phases of its mobilization. The uranium mineralization occurs in association with sulphides and selenides of other metals such as galena, clausthalite, ferroselite, and Ag, Ni and Co selenides.

### Chemical characteristics of investigated material

To make the uranium ores more susceptible to high uranium extraction by leaching, mined ores must be crushed and grounded to produce particles size 0–0.2 mm that can be readily slurried and to expose the uranium minerals to the lixiviant. The chemical composition of the samples of the ores studied was determined in Polish Geological Institute-National Research Institute (PIG-NRI). The basic components of the ores are specified in Table 1.

**Table 1:** The chemical composition of the peribaltic sandstones^a^

	[%]
SiO_2_	22–86
TiO_2_	0.2–0.8
Al_2_O_3_	3–19
Fe_2_O_3_	0.7–10
MnO	0.02–0.4
MgO	0.4–4.4
CaO	0.4–36
Na_2_O	0.3–1.2
SO_3_	<0.01–0.56 (2.8 and 2.6)

^a^Analyses were performed by XRF

The concentration of uranium and accompanying elements was determined by using inductively coupled plasma mass spectrometry (ICP-MS) [17]. The samples represented the material taken from the core of boreholes from Ptaszkowo IG-1 and Krynica Morska IG-3 (Peribaltic Syneclise) were selected by geologists and they can be considered as representative for these specific areas. The analysis of uranium concentration showed the big diversity of uranium concentration in the vertical profile: from 4.2 to 1,316 ppm. Uranium usually was accompanied by other metals, e.g. V, Th, La, Cu or Co. Some of them, which are valuable and occur in significant concentrations can be recovered in technological process to improve the economy of the whole venture. The results of chemical analysis of uranium ore samples that were selected for further leaching tests, are presented in Table 2.

**Table 2:** The content of selected metals in uranium sandstones

Sample notation	Deposit notation	U ppm	V ppm	Th ppm	La ppm	Cu ppm	Co ppm	Fe ppm
21/10/138	Ptaszkowo IG-1	1,120	142	4	14	42	127	8,080
21/10/140	Ptaszkowo IG-1	1,316	625	5.1	47	28	81	12,680
21/10/141	Ptaszkowo IG-1	1,144	717	14	51	47	117	21,590
21/10/142	Ptaszkowo IG-1	670	770	4.8	29	32	57	13,280
21/10/160	Krynica Morska IG-3/I	565	371	4.3	14	78	96	16,000
21/10/161	Krynica Morska IG-3/II	355	158	4.1	25	78	65	13,280
21/10/166	Krynica Morska IG-3/II	260	230	5.3	33	33	35	75,000
21/10/169	Krynica Morska IG-3/II	457	83	7.8	33	65	97	20,780

### Methods

All the leaching experiments were carried out with 0.5 g samples. The investigated material and leaching solution were placed in the round-bottom glass flask equipped with a back cooler and an agitator. The oxidizing agent to oxidize all uranium to U(VI) form, was added. The experiments were performed in the temperature range of 40–80 °C. Furthermore, the influence of other factors such as concentration of the leaching medium, the size of ore particles, liquid to solid mass ratio, temperature, and the type of oxidizing agent, on uranium recovery were also tested. The post-leaching solution was filtered in vacuum and subsequently the ore residues were washed with distilled water. To determine uranium and other elements concentration, known volumes were taken from the post-leaching solution for ICP-MS analyses.

The leaching efficiency was estimated. The mass of residues varied from 50 to 90 % of the mass of starting material. It depended on the type of lixiviant and the mineral composition of ore. When chloric acid was used as a leaching solution the mass of residue was 50–70 %, with sulphuric acid: 80 % and with alkaline solution - 90 %. Each experiment of leaching was repeated 3–5 times in order to confirm the correctness of the obtained results.

The leaching efficiency, *E*, was calculated from the relationship:

$$ {\text{E}} =  \left( {{\text{m/m}}_{\text{o}} } \right)  \times 100\,\%$$

where, m is the total mass of the metal recovered in post-leaching solution and m_o_ is the total mass of the given metal in the ore sample.

## Results and discussion

In the present work various leaching agents were examined e.g. hydrochloric acid, sulphuric acid, a mixture of sodium carbonate and bicarbonate, a mixture of sodium hydroxide and sodium carbonate. Furthermore, the influence of other factors such as concentration of the leaching medium, size of ore particles, liquid to solid ratio, temperature, and the type of oxidizing agent on uranium recovery, was also tested.

### Leaching with hydrochloric and sulphuric acids

**Fig. 3:**
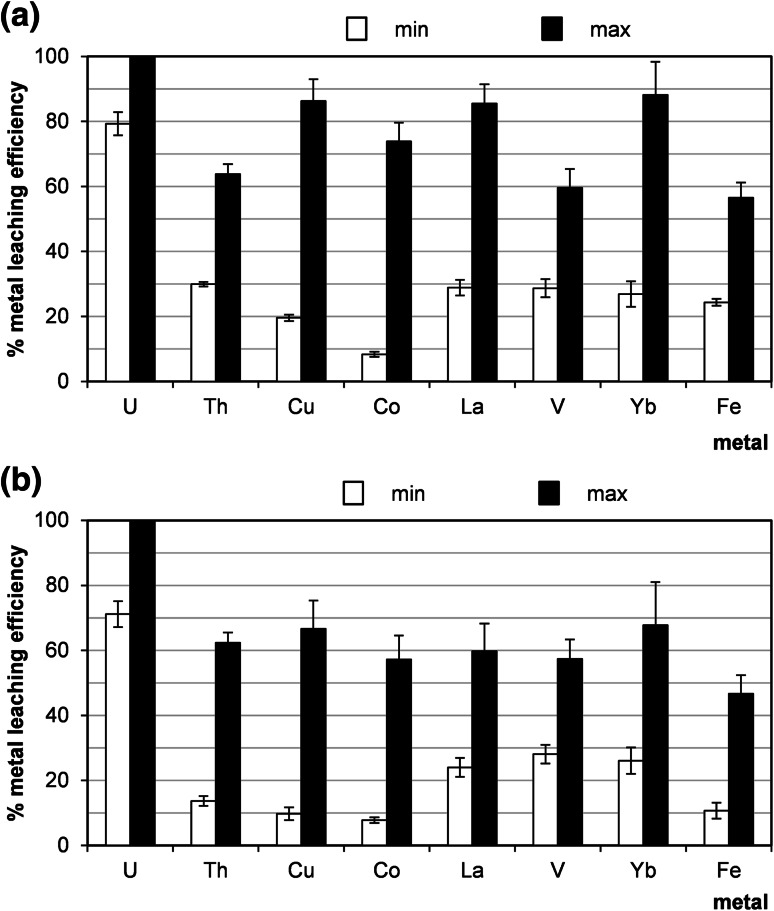
Minimal and maximal values of efficiencies of leaching of metals from sandstones deriving from Peribaltic Syneclise (samples from different boreholes, min: sample 21/10/160, max: sample 21/10/140, see Table 2) under different process conditions: a 10 % HCl, 30 % H_2_O_2_, 60 °C, 1 h, agitation rate: 500 rpm; **b** 10 % H_2_SO_4_, MnO_2_, 60 °C, 1 h, agitation rate: 500 rpm

The uranium recovery with 10 % hydrochloric acid at 60 °C was very efficient (Fig. 3a). The results of experiments revealed that extraction efficiency of uranium range from 79 to almost 100 %. The other metals were recovered with efficiencies, as follows: Th: 30–64 %, Cu: 20–86 %, Co: 8–74 %, La: 29–86 %, V: 29–60 %, Yb: 27–88 %, Fe: 25–56 %. Experiments with 10 % sulphuric acid as leach agent was carried out at 60 °C. As it can be observed in Fig. 3b, uranium was leached with efficiency 71–100 % and efficiencies of leaching other metals were: Th:13–62 %, Cu 10–67 %, Co: 8–57 %, La: 24–60 %, V: 28–58 %, Yb: 26–67 %, Fe: 11–47 %.

The leaching efficiencies of uranium and metals accompanying it vary in a broad range. Interpretation is rather difficult because as was said earlier, the effect of ore mineralogy and mineral liberation on the leaching behaviour of uranium and other metals is not well defined.

**Fig. 4:**
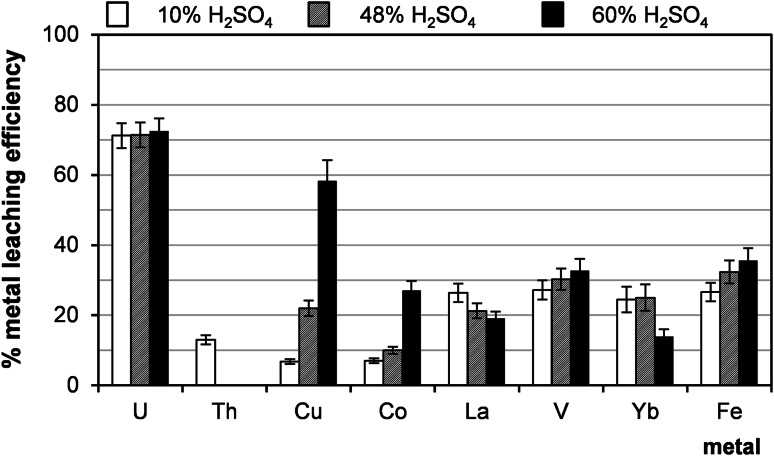
Effect of sulphuric acid concentration on leaching efficiency (sample 21/10/160). Process conditions: particle size: 0–0.2 mm, liquid/solid ratio of 8:1 (vol./wt. basis), oxidizing agent MnO_2_, 60 °C, 1 h, agitation rate: 500 rpm

In order to examine the effect of sulphuric acid concentration on leaching efficiency, a series of experiments was performed in such a way that the concentration of sulphuric acid was changed from 10 to 48 % (Fig. 4). It was concluded that acid concentration had no influence on the uranium leaching but it influenced the lanthanide leaching. The efficiency of leaching decreased with the increasing sulphuric acid concentration. The results obtained in these investigations are in agreement with the knowledge about solubility of lanthanum sulphates in aqueous solutions [18].

**Fig. 5:**
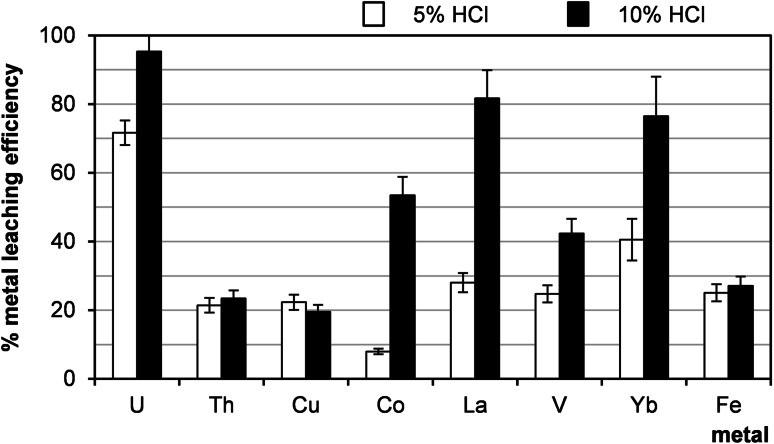
Effect of hydrochloric acid concentration on leaching efficiency (sample 21/10/160). Process conditions: particle size: 0–0.2 mm, liquid/solid ratio of 8:1 (vol./wt. basis), oxidizing agent: H_2_O_2_, 60 °C, 1 h, agitation rate: 500 rpm

In contrast to the results above, a very significant increase in the leaching efficiency of uranium and accompanying metals with the concentration of hydrochloric acid was observed, as it is presented in Fig. 5. Lanthanum chlorides in contrast to the sulphates are highly soluble in water.

### Leaching with alkaline carbonate solutions

**Fig. 6:**
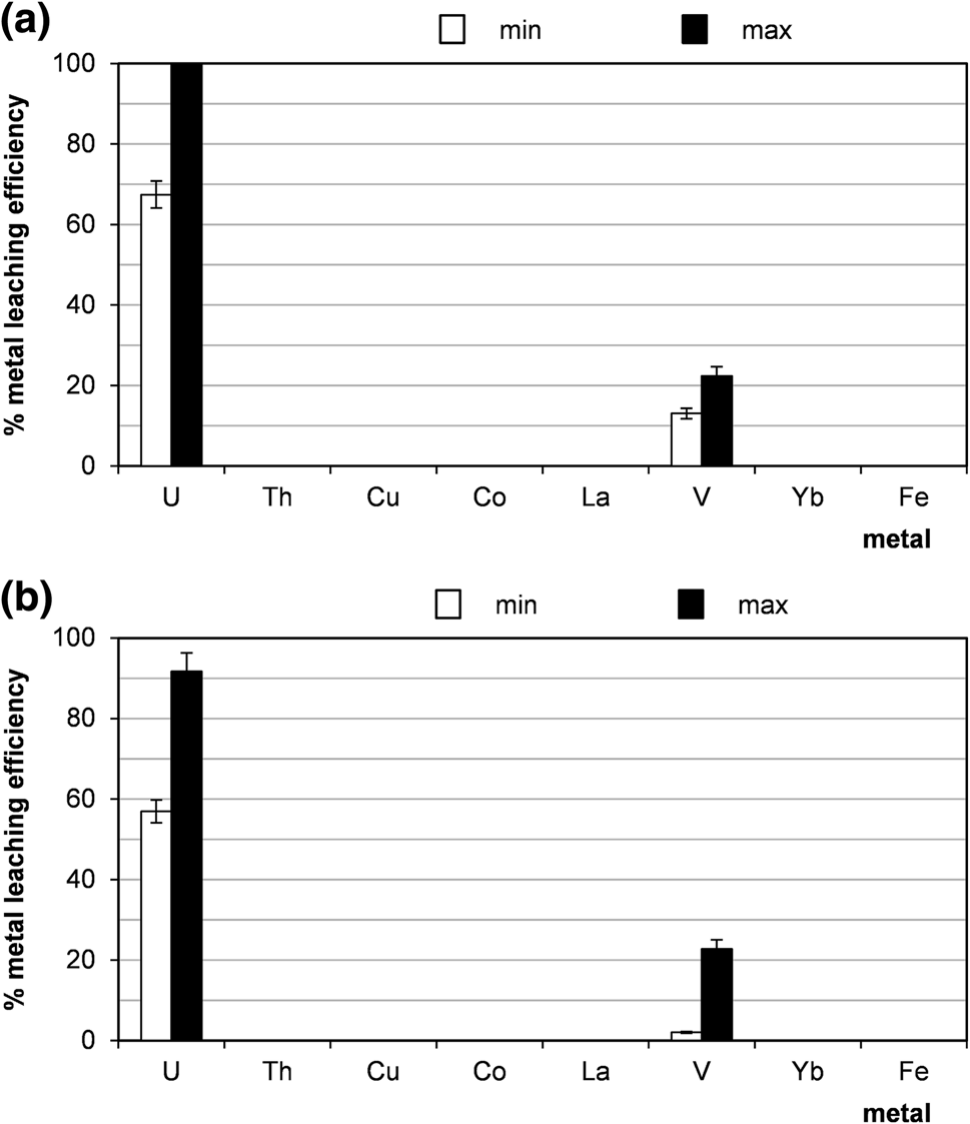
Minimal and maximal values of efficiency of leaching of metals from sandstones deriving from Peribaltic Syneclise (samples from different boreholes, min: sample 21/10/160, max: sample 21/10/140, see Table 2) under different process conditions: **a** 8 % NaOH/18 % Na_2_CO_3_, 30 % H_2_O_2_, 60 °C, 1 h, agitation rate: 500 rpm, **b** 5 % Na_2_CO_3_/NaHCO_3_, KMnO_4_, 60 °C, 1 h, agitation rate: 500 rpm

As it was mentioned earlier, the alkaline leaching is highly selective for uranium. In present experiments, more than 80 % of uranium was leached with a mixture of sodium carbonate and bicarbonate (Na_2_CO_3_/NaHCO_3_) containing KMnO_4_ (Fig. 6a) and almost 100 % of uranium was leached with alkaline carbonate solution (8 % NaOH/18 % Na_2_CO_3_) containing H_2_O_2_ (Fig. 6b). In post-leaching solutions only uranium and vanadium were detected.

### Effect of leaching time

**Fig. 7:**
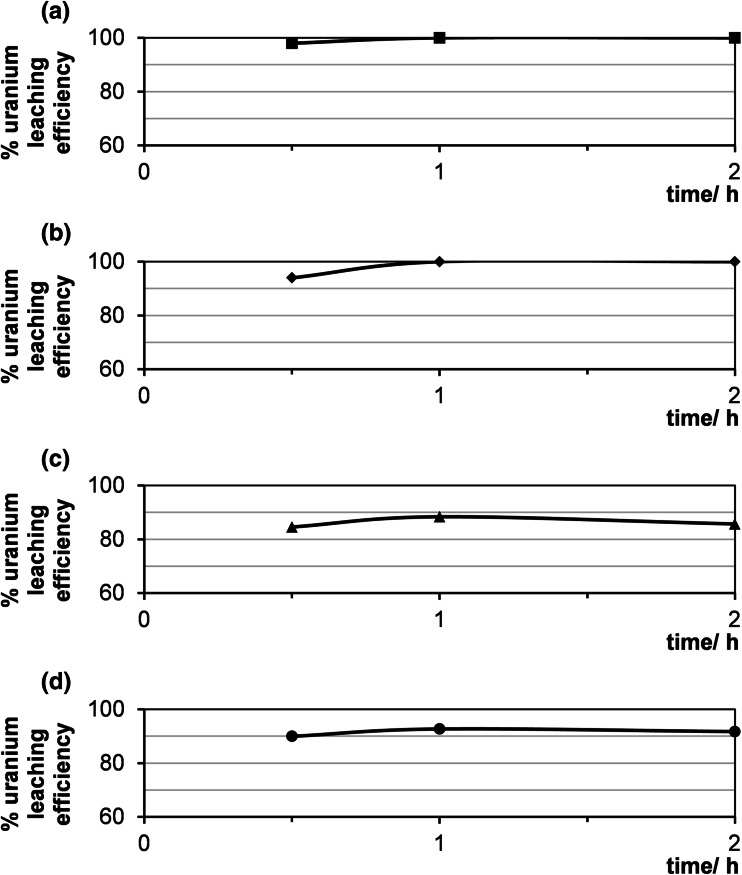
Effect of time on uranium leaching efficiency (sample 21/10/140). Process conditions: particle size: 0–0.2 mm, liquid/solid ratio of 8:1 (vol./wt. basis), 60 °C, agitation rate: 500 rpm, lixiviants: **a** 10 % HCl **b** 10 % H_2_SO_4_, **c** 8 % NaOH/18 % Na_2_CO_3_ **d** 5 % Na_2_CO_3_/NaHCO_3_

Leaching time should be experimentally chosen based on the characteristics of the ore e.g. type of mineralization, particle size and leaching conditions. In this work, the time of leaching was changed from 0.5 to 2 h in a series of tests under the same conditions. As it can be seen in Fig. 7, after 1 h no increase of uranium leaching efficiency was observed. Thus 1 h was the optimum leaching time.

### Effect of temperature

**Fig. 8:**
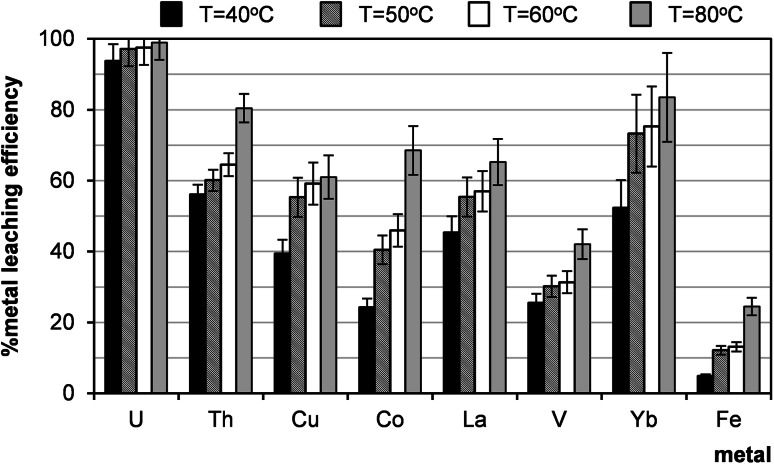
Effect of temperature on leaching efficiency (sample 21/10/166). Process conditions: particle size: 0–0.2 mm, 10 % H_2_SO_4_, liquid/solid ratio of 8:1 (vol./wt. basis), oxidizing agent: MnO_2_, 1 h, agitation rate: 500 rpm

Temperature is an important factor playing a significant role in the leaching process. In the present study the efficiency of uranium leaching increased slightly with the range of 40–80 °C (Fig. 8). However, the efficiencies of leaching of other metals increased by 20–40 % along with an increase of temperature. Hence, there was found that the optimal temperature of leaching process of all metals was 60 °C.

### Effect of solid to liquid ratio

**Fig. 9:**
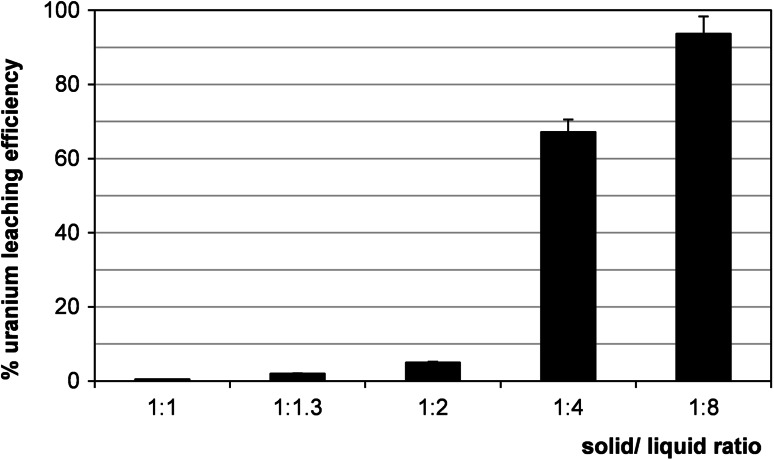
Effect of liquid/solid ratio (vol./wt. basis) on uranium leaching efficiency (sample 21/10/141). Process conditions: particle size: 0–0.2 mm,10 % H_2_SO_4_, oxidizing agent: MnO_2_, 60 °C,1 h, agitation rate: 500 rpm

The selection of proper solid to liquid ratio (the weight of ores to volume of lixiviant) is important for optimization the leaching process. The effect of the solid to liquid ratio depends on e.g. grain distribution and free surface [19]. For this reason it was necessary to test the effect of solid to liquid ratio on leaching operations of Polish sandstones. This effect was significant as it shown on Fig. 9. The yield of uranium extraction increased with decrease of density of slurries. The optimum solid to liquid ratio was found to be 1:8.

### Effect of particle size

**Fig. 10:**
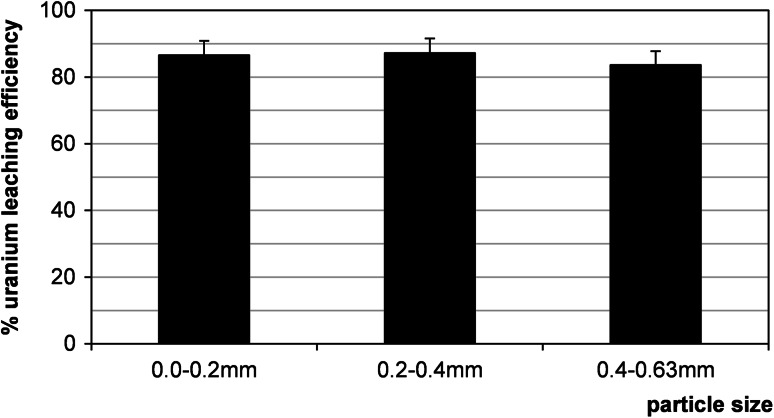
Effect of particle size on uranium leaching efficiency (sample 21/10/161). Process conditions: 10 % H_2_SO_4_, MnO_2_, liquid/solid ratio of 8:1 (vol./wt. basis), 60 °C, 1 h, agitation rate: 500 rpm

The influence of particle size of ore on the efficiency of uranium leaching process was studied using 10 % H_2_SO_4_/MnO_2_ system at 60 °C with a liquid/solid ratio 8:1 (vol./wt. basis) for 21/10/161 sample. Three fractions with different granulations were tested: 0–0.2 mm, 0.2–0.4 and 0.4–0.63 mm. Under above conditions, no evident influence of particle size on the metal leaching efficiencies was observed (Fig. 10). Usually, the literature shows that the decrease in particle size enhanced metals dissolution. The observed small differences can be likely ascribed to the mineralogical and elemental distribution within the sizes and interaction of the minerals/phases within the ore [19].

### Effect of oxidation agent

**Fig. 11:**
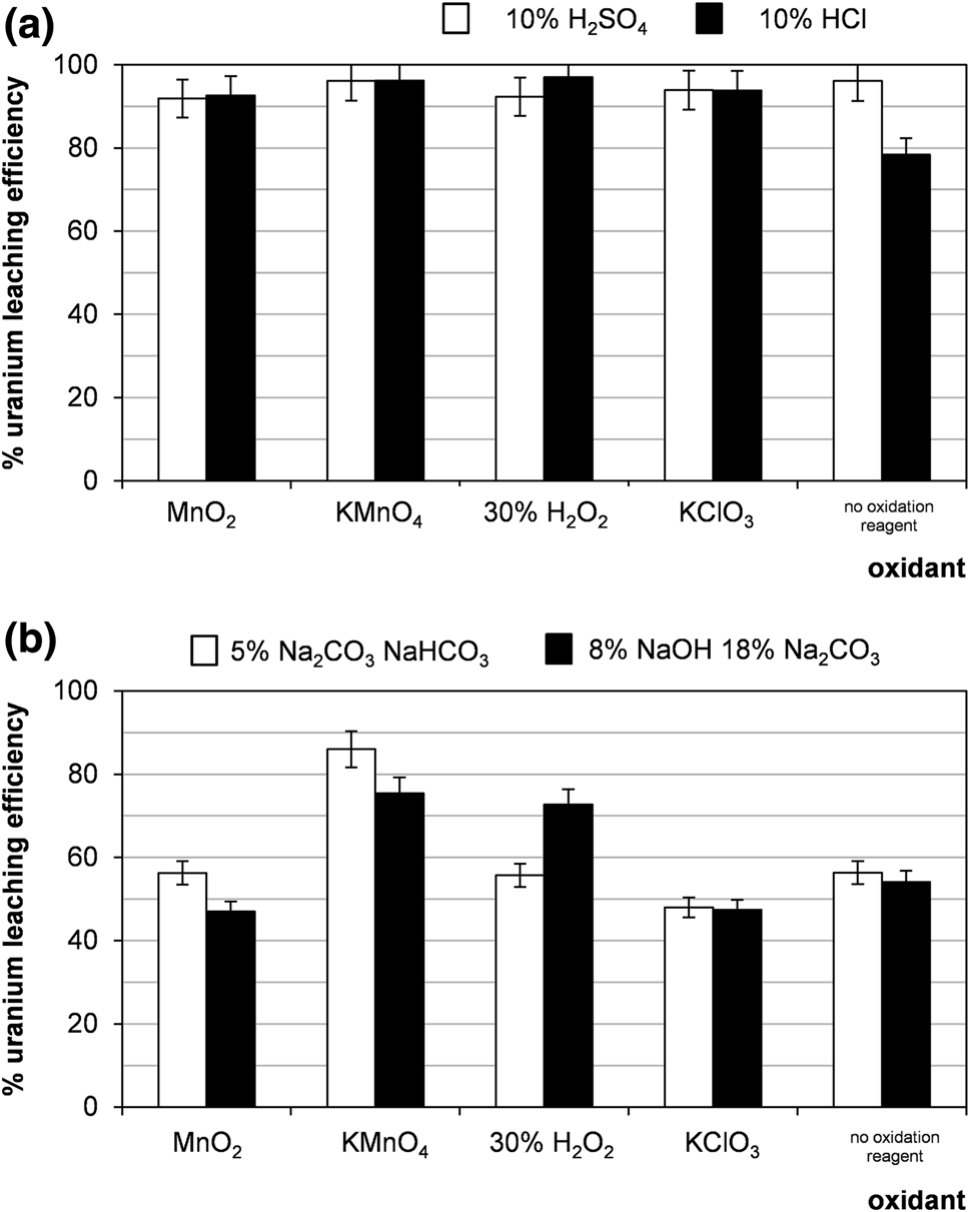
Effect of oxidizing agent on uranium leaching efficiency (sample 21/10/169). Process conditions: **a** particle size: 0–0.2 mm, 60 °C, 1 h, agitation rate: 500 rpm, **b** particle size: 0–0.2 mm, 60 °C, 1 h, agitation rate: 500 rpm

The first step in any uranium leaching process is the oxidation of insoluble U^4+^ to soluble U^6+^ oxidation state. In the present work various oxidizing agents e.g. MnO_2_, KMnO_4_, H_2_O_2_, KClO_3_ were tested. In acid leaching significant difference in effectiveness of oxidizing agent was not observed (Fig. 11a). In alkaline leaching, KMnO_4_ and H_2_O_2_ were found as the most efficient oxidizers (Fig. 11b).

The leaching process without the addition of the oxidizing agent was also examined. This method works only for the uranium deposits that are rich in iron, especially in Fe^3+^ form. It is due to that the ferric ion actually oxidizes uranium, while the oxidizing agent oxidizes ferrous ion to ferric ion in accordance with the scheme:

$$ {\text{Fe}}^{ 2+ } + {\text{ MnO}}_{ 2} + 4 {\text{H}}^{ + } \to {\text{Fe}}^{ 3+ } + {\text{Mn}}^{ 2+ } + {\text{ 2H}}_{ 2} {\text{O}} $$

$$ {\text{UO}}_{ 2} + 2 {\text{Fe}}^{ 3+ } \to {\text{UO}}_{ 2}^{ 2+ } + 2 {\text{Fe}}^{ 2+ } $$

**Fig. 12:**
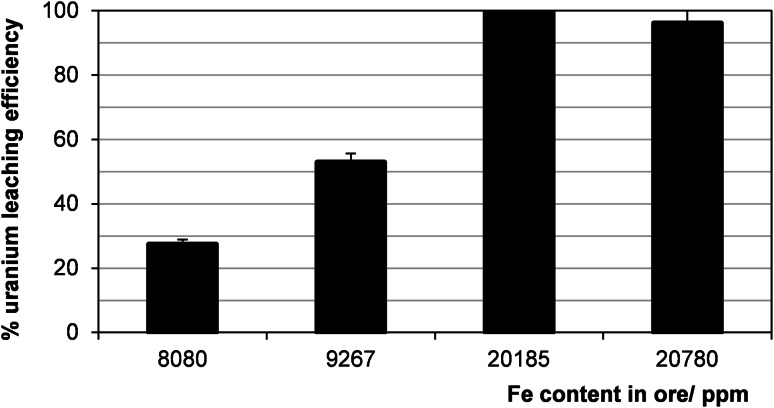
Effect of concentration of iron in ore on uranium leaching efficiency. Process conditions: particle size: 0–0.2 mm, 10 % H_2_SO_4_, liquid/solid ratio of 8:1 (vol./wt. basis), 60 °C, 1 h, agitation rate: 500 rpm

The efficiencies of uranium leaching with 10 % H_2_SO_4_ without the oxidizing agent with reference to concentration of iron in the ore is showed on Fig. 12.

### Leaching with water, aq. NH_4_Cl and HNO_3_

**Fig. 13:**
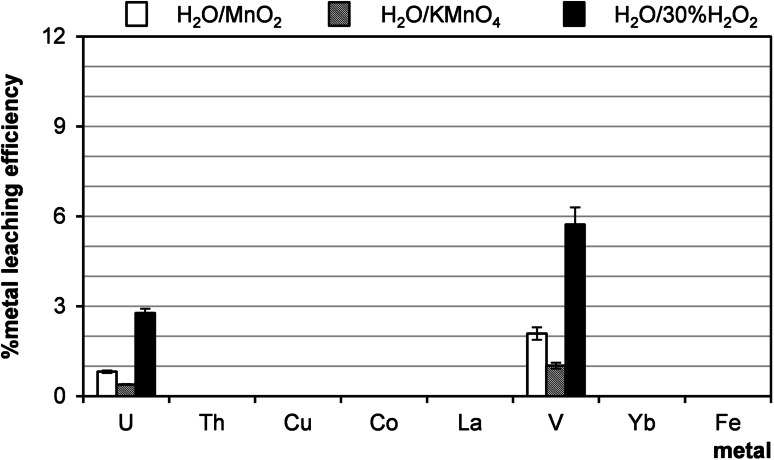
Metal extraction behavior with distilled water and oxidants. Process conditions: particle size: 0–0.2 mm, liquid/solid ratio of 8:1 (vol./wt. basis), 60 °C, 1 h, agitation rate: 500 rpm

**Fig. 14:**
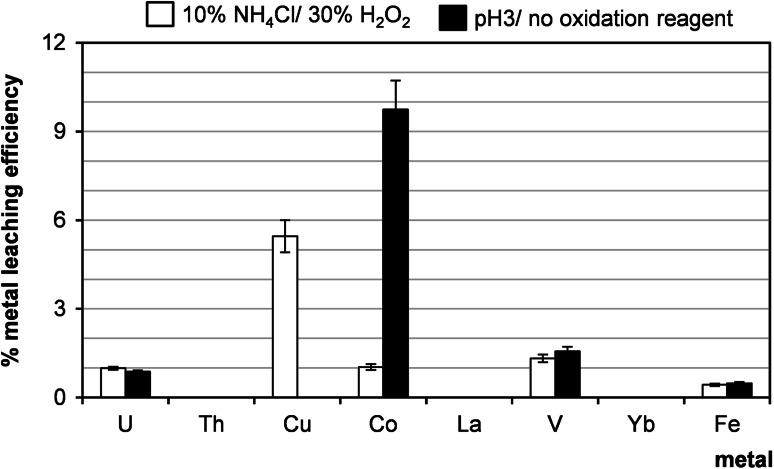
Metal extraction behavior with 10 % NH_4_Cl/H_2_O_2_ and HNO_3_, pH 3, without oxidants. Process conditions: particle size: 0–0.2 mm, liquid/solid ratio of 8:1 (vol./wt. basis), 60 °C, 1 h, agitation rate: 500 rpm

The results of leaching uranium ores with distilled water in the presence of oxidants are presented in Fig. 13. This method proved not to be suitable for leaching uranium from Polish sandstones. Also leaching with aq. NH_4_Cl/H_2_O_2_ and aq. HNO_3_ (pH 3) was not effective (Fig. 14).

## Conclusion

These studies showed that it was very difficult to achieve uranium extraction higher than 90 % for Triassic Peribaltic sandstones under conventional leaching conditions. Many factors such as, ore mineralogy, the type of leaching medium and its concentration, size of particles of solid material, temperature, type of oxidizing agent, and liquid to solid ratio, influence uranium recovery process.

In the first experiments the influence of time on leaching process was examined and it was concluded that 1 h is sufficient for uranium extraction. In comparison with acid processing, alkaline leaching had the advantage of being selective for uranium. The all metals accompanying uranium in the ores were also present in acid post-leaching solutions. In the case of alkaline leaching process only two metallic components of the ores were detected: U and small amounts of V.

In the temperature range from 40 to 80 °C, the significant change of the efficiency of uranium leaching was not observed. However, increase of temperature enhances the extraction of other metals. The optimal process temperature was 60 °C. It is very important that it is not necessary to ground ores below 0.63 mm because the influence of particle size on the metal leaching efficiencies was not observed up to this particle size.

In acid leaching the significant difference in effectiveness of oxidizing agent (MnO_2_, KMnO_4_, H_2_O_2_, KClO_3_) was not observed. In another way, in alkaline leaching, KMnO_4_ and H_2_O_2_ were found as the most efficient oxidizers.

The solid–liquid extraction is a very important stage in the technology of uranium production from the uranium ores. It is important to extract at this stage as large as possible amount of the metals, which are of interest for the economy reasons. Appropriate selection of the parameters allows controlling the process efficiency. Apart from uranium such components of the ores like molybdenum, vanadium or rare earth elements are considered for recovery. The selectivity of the leaching stage to some components by appropriate selection of reagents gives more flexibility in process design and further arrangement of technological flow sheet. The recovery of not only uranium but also other valuable metals could to be considered in the technological scheme to improve economy of such a project.

The solid–liquid extraction is the first stage of the technology of uranium production that is followed by other steps: purification-concentration by ion exchange resins or liquid–liquid extraction/re-extraction and precipitation to obtain final product, yellow cake U_3_O_8_.
